# Deep-Reinforcement-Learning-Based Object Transportation Using Task Space Decomposition

**DOI:** 10.3390/s23104807

**Published:** 2023-05-16

**Authors:** Gyuho Eoh

**Affiliations:** Department of Mechatronics Engineering, Tech University of Korea, 237 Sangidaehak-ro, Siheung-si 15073, Gyeonggi-do, Republic of Korea; gyuho.eoh@tukorea.ac.kr; Tel.: +82-31-8041-0468

**Keywords:** object transportation, deep reinforcement learning, task space decomposition

## Abstract

This paper presents a novel object transportation method using deep reinforcement learning (DRL) and the task space decomposition (TSD) method. Most previous studies on DRL-based object transportation worked well only in the specific environment where a robot learned how to transport an object. Another drawback was that DRL only converged in relatively small environments. This is because the existing DRL-based object transportation methods are highly dependent on learning conditions and training environments; they cannot be applied to large and complicated environments. Therefore, we propose a new DRL-based object transportation that decomposes a difficult task space to be transported into simple multiple sub-task spaces using the TSD method. First, a robot sufficiently learned how to transport an object in a standard learning environment (SLE) that has small and symmetric structures. Then, a whole-task space was decomposed into several sub-task spaces by considering the size of the SLE, and we created sub-goals for each sub-task space. Finally, the robot transported an object by sequentially occupying the sub-goals. The proposed method can be extended to a large and complicated new environment as well as the training environment without additional learning or re-learning. Simulations in different environments are presented to verify the proposed method, such as a long corridor, polygons, and a maze.

## 1. Introduction

Object transportation is where a robot manipulates an object to a goal, and it is applied to various fields, such as logistics [1,2], exploration [3,4], retrieval tasks [5], and foraging [6].

Representative methods of transporting an object are prehensile and non-prehensile manipulation [7]. In prehensile manipulation, a robot first firmly grasps or captures an object using an equipped tool such as a gripper [8] or a dexterous robotic hand [9]. The robot and the object then move toward a goal as one body. The advantage of prehensile manipulation is that the object remains stationary during the transportation process, which allows the robot to precisely control the object. However, the disadvantage is that a pre-gripping or capturing motion is required before the object is transported. In addition, a robot should carefully determine how to grasp or where to apply force depending on the shape and material of the object. In contrast to prehensile manipulation, in non-prehensile manipulation, a robot does not use any tools and only pushes an object with its body [10,11,12]; preliminary grasping actions for object transportation are not required. In addition, the control of the robot is easier than that of prehensile manipulation because the robot is not tied to an object. However, it is difficult to predict the motion of the object, and thus a robot should reposition its pushing point every second to transport an object to a goal. Because of these characteristics, prehensile manipulation is an appropriate method for structured objects (i.e., known objects) [13] and non-prehensile manipulation is applicable to unstructured objects (i.e., unknown objects) [14,15].

Recently, reinforcement learning (RL)-based non-prehensile manipulation methods have been studied by many researchers [16,17,18,19]. These methods have performed well by learning how to transport an object without external intervention, but they only worked in limited environments (i.e., trained environments). In other words, their methods did not work well when the test and learning environments were different. In order to operate in a new environment, a robot would have to be retrained; this is an inefficient method due to time and space constraints.

Therefore, we propose a novel object transportation using deep reinforcement learning (DRL) and task space decomposition (TSD). First, a robot learns how to transport an object in a standard learning environment (SLE). The SLE is a learning space with a symmetric structure, which is usually smaller than a test environment, that allows a robot to learn a basic policy required for object transportation. In this phase, the robot becomes proficient in transporting an object using DRL. Second, a test environment (i.e., a whole task space) is divided into multiple sub-task spaces by considering the maximum traveled distance of a robot, which is called the TSD method. The TSD method divides a large and complex environment into many small and simple environments, making it easier to transport objects step-by-step. Finally, the object is transported to the goal by following the policy learned from the SLE in sub-task spaces. Each sub-task space computed from the TSD is smaller than the SLE, and thus the robot can apply the policy learned in the SLE. In other words, we simplified the problem by extending the learning results in the sub-task space to the whole-task space, as opposed to the existing DRL-based object transportation in the whole environment at once (i.e., end-to-end learning); this is the main contribution of this paper.

The proposed method has retained the advantage of prehensile manipulation, where a robot can learn how to transport without the complexity of control. At the same time, it trains only in a small environment (i.e., SLE), where it is easy to learn by DRL, and solves problems in large environments using the results in the SLE. This is similar to the divide and conquer strategy in computer science [20], and it has the advantage of being applicable to a wide variety of environments.

The contributions of this paper are summarized as follows:

- We present a novel object transportation method using DRL and TSD.
- We propose a DRL-based object transportation training method within an SLE.
- A TSD approach is introduced that can be applied to arbitrary object transportation environments.
- We verify the proposed method by performing simulations in a variety of environments, such as a long corridor, a polygon, and a maze.

This paper is organized as follows. Section 2 describes DRL-based non-prehensile object transportation methods and task decomposition methods. The object transportation problem is defined in Section 3, and the system overview is presented in Section 4. Section 5 and Section 6 explain the proposed object transportation method by separating the training and TSD steps. Simulations are shown in Section 7, and the conclusion is presented in Section 9.

## 2. Related Work

Traditionally, RL has been used as a tool to solve the object transportation problem and has been studied by many researchers. Manko et al. [21] presented a Q-learning-based large-object transportation method in a complex environment. Wang and De Silva [22] compared a single-agent Q-learning with a team Q-learning. They showed that single-agent Q-learning is better than the team Q-learning in a complicated environment because of the local-minima. Similarly, Rahimi et al. [23] compared different Q-learning-based box-pushing algorithms, and Wang and de Silva [24] proposed sequential Q-learning with the Kalman filtering method.

The above-mentioned RL-based object transportation methods showed limited performance under certain conditions because traditional Q-learning algorithms (e.g., the tabular method) have finite possible states. In the real-world, the possible states are very large, and thus a robot cannot sufficiently learn how to transport due to the limited size of the Q-table. Therefore, Mnih et al. [25] and Silver et al. [26] presented a deep Q-network (DQN) algorithm that shows a good performance by approximating the Q-table with a deep neural network.

Based on the improved performance of DQN, many DRL-based object transportation studies have been proposed. Zhang et al. [27] proposed multi-robot object transportation based on DRL. A large rod is carried to an exit by two robots using DQN. Xiao et al. [28] presented a decentralized macro-action-based policy via a centralized Q-net. Each decentralized Q-net is trained with the help of a centralized Q-net. Eoh and Park [29] proposed a curriculum-learning-based object transportation method using difficulty map generation and an adaptive determination of the episode size. Shibata et al. [30] presented a DRL-based multi-robot transportation method using an event-triggered communication and consensus-based control. The proposed multi-robot team can balance the control performance and communication savings even when the number of agents is different from that in the learning environment. In addition, various manipulation skills (e.g., pushing, pulling, and moving objects) based on RL have been studied in the fields of animation and virtual reality [31,32]. Their proposed methods allow a producer to create transport animations without complicated and tricky editing.

Meanwhile, various task decomposition methods have been proposed by many researchers to simplify complex navigation tasks. Kawano [33] proposed an automatic hierarchical sub-task decomposition method for a multi-robot delivery mission. They decomposed a task into multiple sub-tasks and learned in a stepwise manner by expanding the activity radius of the robot. This is a similar method to our proposed method, but their method only works in a grid world; robots have limited movements and action policies, such as pushing, non-pushing, and waiting. Mesesan et al. [34] proposed a hierarchical path planner that consists of two stages: a global planner and several local planners. It solves a complicated global path-planning problem by decomposing it into simpler local path-planning problems. Zhang et al. [35] proposed task-space-decomposed motion planning. The constrained manifold can be easily solved by a dual-resolution motion planning framework consisting of a global planner and a local planner. Fosong et al. [36] presented a multi-agent teamwork-learning method using a sub-task curriculum. A multi-agent learns simple specific sub-tasks, and then the policy of the team is merged and transferred to the target task. The above-mentioned task decomposition methods have different methodologies, but they all have one thing in common: they solve problems by breaking down a difficult problem into multiple easy problems. Following this principle, we propose a new object transportation approach that applies the learning results in a small and simple SLE to large and complex environments.

## 3. Problem Description

The problem of object transportation is manipulating an object to a goal within the goal boundary $\epsilon_{success}$ using a robot as follows:

(1) $$\left|\right|p_{t}^{o}-p^{g}{\left|\right|}_{2}<\epsilon_{success}$$

where $p_{t}^{o}$ is the position of the object at time *t* and $p^{g}$ is the position of the goal. If an object is within $\epsilon_{success}$, the object transport is considered complete. The robot is able to manipulate an object by pushing under its own power. We assumed that the object follows the quasi-static model, which is a reasonable assumption except for when the object is spherical. The environment was assumed to be polygonal in shape and, if it is not, it can be approximated as a polygon. We also assumed that there are no obstacles in the environment in order to focus on how an object is transported.

## 4. System Overview

The proposed object transportation method is based on DRL and TSD. This process is performed in three steps: (1) training to transport an object in an SLE, (2) TSD and sub-goal generation, and (3) transporting an object via sub-goals in the whole-task space.

**Figure 1:**
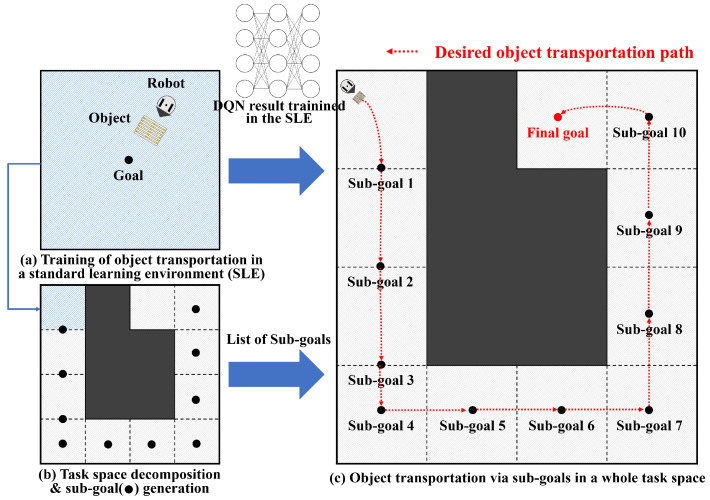
Overview of DRL-based object transport using the TSD method. (**a**) A robot learns how to transport an object to a goal in an SLE. A deep Q-network (DQN) is used for training. (**b**) A whole-task space is decomposed into sub-task spaces by considering the size of the SLE. The size of the sub-task spaces cannot exceed the size of the SLE. For each sub-task space, sub-goals are generated by considering the maximum traveled distance of a robot. (**c**) The object is transported via the sub-goals by the pushing motion of a robot.

First, we created the SLE in a small and square shape to facilitate learning, as shown in Figure 1a. In the training phase, we initialized the robot’s position randomly but ensured that the goal position is always in the center of the environment for symmetry. The size of the SLE can vary, but there are tradeoffs, such as the following. If the SLE is small, it is easy to converge and fast to learn, but the whole-task space should be divided into many small pieces in the transportation phase. On the other hand, if the SLE is large, then the number of sub-goals will decrease because the space of the sub-tasks will also be large. However, it is prone to divergence and the learning speed is slow.

Second, a whole-task space was decomposed into the spaces of sub-tasks, taking into account the size of the SLE in the first phase, as shown in Figure 1b. The size of the sub-tasks cannot exceed that of the SLE because a robot cannot learn how to transport an object beyond the size of the SLE. Several sub-goals were then computed, taking into account the shape of the environment and the size of the sub-task spaces.

Finally, a robot transported an object to a goal via sub-goals that were generated in the previous phase, as shown in Figure 1c. For each space of sub-tasks, the robot takes an action by applying the inference result of the DQN, which was pre-trained in the SLE. The robot can iteratively use the pre-trained results by sequentially changing a goal from the initial to the final sub-goal. For example, in Figure 1c, the robot selects a goal as sub-goal 1 at the beginning. When the robot has successfully transported an object to sub-goal 1, a goal is changed to sub-goal 2. The robot then transports an object to sub-goal 2. This continues until the object is successfully transported to the final goal.

## 5. Training in a Standard Learning Environment

In this section, we describe the training process for object transportation in an SLE. First, a Markov decision process (MDP) was introduced to solve the RL problem. Next, the DRL framework for object transportation is presented and each component of the framework is described. Finally, an object transportation training method based on DQN is proposed for efficient learning.

### 5.1. Markov Decision Process

The RL problem can be solved by finding sequential decisions under uncertainty, which was formulated by the MDP. The MDP consists of four tuples: state space (S), action space (A), state transition probability (T), and reward function (R). A robot observes the state $s_{t}\in S$ at time *t* and takes an action $a_{t}\in A$ according to a policy function $\pi :s_{t}\to Prob\left(A|s_{t}\right)$. The policy maps states to actions and determines how the robot interacts with the environment. The state transition probability $T\left(s,a\right)=Prob\left(s_{t+1}=s^{\prime }|s_{t}=s,a_{t}=a\right)$ is the probability when a robot takes the action *a* at the state *s*. A robot receives a reward $r_{t}\in R:S\times A\to R$ if a robot performs an action $a_{t}$ in a given state $s_{t}$. The goal of RL is to find a policy function π that maximizes the sum of all expected rewards over time.

### 5.2. Reinforcement Learning Framework for Object Transportation

**Figure 2:**
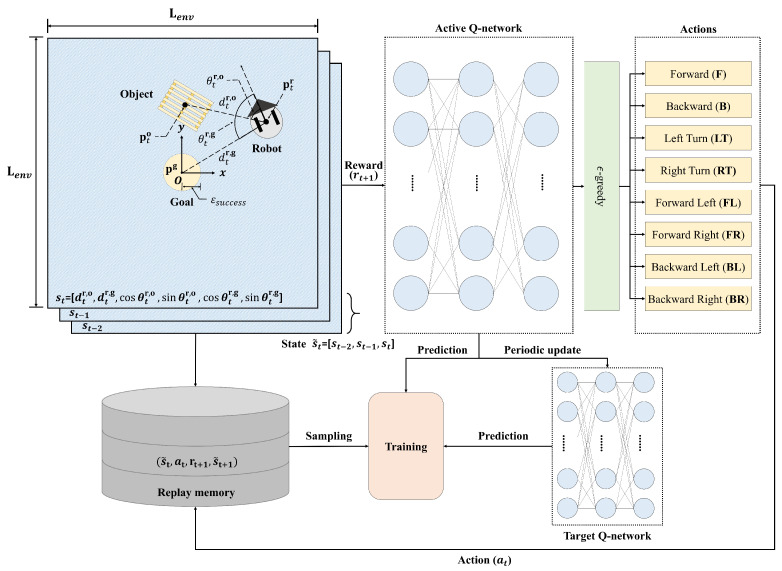
Object transport training process in a square SLE.

An RL framework for object transportation is presented in Figure 2. A square environment ($L_{env}\times L_{env}$) was created for standardized learning. The initial positions of a robot and an object were chosen randomly. However, the goal position should be fixed in the center (i.e., origin) of the SLE to preserve symmetry: $p^{g}=\left[0,0\right]$ and $p_{t}^{r},p_{t}^{o}\in \left[-\frac{L_{env}}{2},\;\frac{L_{env}}{2}\right]\times \left[-\frac{L_{env}}{2},\;\frac{L_{env}}{2}\right]$.

A state $s_{t}$ consists of a six-dimensional array as follows:

(2) $$\begin{array}{c} s_{t}=\left[d_{t}^{r,o},\;d_{t}^{r,g},\;\cos\theta_{t}^{r,o},\;\sin\theta_{t}^{r,o},\;\cos\theta_{t}^{r,g},\;\sin\theta_{t}^{r,g}\right], \end{array}$$

where $d_{t}^{i,j}$ and $\theta_{t}^{i,j}$ are the distance and the angle between *i* and *j*, respectively. The indices *i* and *j* can represent a robot, an object, and a goal: i,j∈{robot,object,goal}. In order to represent the robot’s pushing motion, we defined a new state by concatenating the last three consecutive frames similar to [25]:

(3) $$\begin{array}{c} {\tilde{s}}_{t}=concatenate\left\{s_{t-2},s_{t-1},s_{t}\right\}. \end{array}$$

The actions of the robot are represented by eight motions using translational and rotational velocities ($\left[v_{trans},v_{rot}\right]$) as follows:

(4) $$\begin{array}{c} a_{t}=\left\{\begin{array}{cc} \left[-v_{trans},0\right] & Backward(B)\\ {}[v_{trans},0] & Forward(F)\\ {}[0,-\frac{v_{rot}}{2}] & Left\;Turn(\mathrm{LT})\\ {}[0,\frac{v_{rot}}{2}] & Right\;Turn(\mathrm{RT})\\ {}[v_{trans},-v_{rot}] & Forward\;Left(\mathrm{FL})\\ {}[v_{trans},v_{rot}] & Forward\;Right(\mathrm{FR})\\ {}[-v_{trans},-v_{rot}] & Backward\;Left(\mathrm{BL})\\ {}[-v_{trans},v_{rot}] & Backward\;Right(\mathrm{BR}) \end{array}\right. \end{array}$$

A robot receives a reward according to action $a_{t}$ given the state ${\tilde{s}}_{t}$ as follows:

(5) $$\begin{array}{c} r_{t}\left({\tilde{s}}_{t},a_{t}\right)=\left\{\begin{array}{cc} r_{success} & \left|\right|p_{t}^{o}-p^{g}{\left|\right|}_{2}<\epsilon_{success}\\ 2r_{collision} & \left|\right|p_{t}^{o}-p^{g}{\left|\right|}_{1}>\frac{L}{2}-\epsilon_{margin}\\ r_{collision} & \left|\right|p_{t}^{r}-p^{g}{\left|\right|}_{1}>\frac{L}{2}-\epsilon_{margin}\\ w_{1}\Delta d_{t}^{g,o}+w_{2}\Delta d_{t}^{r,o} & \mathrm{otherwise} \end{array}\right. \end{array}$$

where $r_{success}>0$ and $r_{collision}<0$ are rewards according to the success of object transportation and the collision with respect to the wall, respectively. The margin parameter $\epsilon_{margin}$ is determined by considering the size of the SLE, a robot, and an object. The indicator function $\Delta d_{t}^{i,j}$ is how close an agent *i* is getting to an agent *j* (i,j∈{robot,object,goal}). Since it is most important that the object reaches its goal, we set $w_{1}$ and $w_{2}$ to satisfy the following conditions: $w_{1}=10w_{2}$.

### 5.3. Object Transportation Learning Using Deep Q-Network

Q-learning is a model-free RL that uses the Bellman equation to iteratively update estimates of the expected rewards of each action, allowing an agent to learn an optimal policy that maximizes the long-term expected return [37]. This process is formulated as a Q-learning algorithm:

(6) $$\begin{array}{c} Q\left(s_{t},a_{t}\right)\leftarrow Q\left(s_{t},a_{t}\right)+\alpha \left[r_{t+1}+\gamma \max_{a_{t+1}}Q\left(s_{t+1},a_{t+1}\right)-Q\left(s_{t},a_{t}\right)\right], \end{array}$$

where α∈(0,1] is a learning rate that determines how fast a robot will learn. If α≈0, a robot will not learn anything. On the contrary, a robot will ignore previous information if α=1. Parameter γ∈[0,1] is the discount factor that determines how much future rewards will be considered. If γ=1, a robot will consider a long-term reward. Conversely, a robot will only consider a short-term (i.e., immediate) reward if γ=0.

The Q-table generated by the Q-learning algorithm can be parameterized by a neural network denoted as $\theta_{t}$. We can rewrite Equation (6) as follows:

(7) $$\begin{array}{c} Q\left(s_{t},a_{t}|\theta_{t}\right)\leftarrow Q\left(s_{t},a_{t}|\theta_{t}\right)+\alpha \left[r_{t+1}+\gamma \max_{a_{t+1}}Q\left(s_{t+1},a_{t+1}|\theta_{t}^{\prime }\right)-Q\left(s_{t},a_{t}|\theta_{t}\right)\right], \end{array}$$

where $\theta_{t}$ and $\theta_{t}^{\prime }$ are the weights and biases in the active and target Q-networks, respectively. The active and target Q-networks are illustrated in Figure 2.

The loss function $L(s_{t},a_{t}|\theta_{t})$ is defined as follows:

(8) $$\begin{array}{c} L\left(s_{t},a_{t}|\theta_{t}\right)\leftarrow {\left[r_{t+1}+\gamma \max_{a_{t+1}}Q\left(s_{t+1},a_{t+1}|\theta_{t}^{\prime }\right)-Q\left(s_{t},a_{t}|\theta_{t}\right)\right]}^{2}, \end{array}$$

and the weights and biases are updated to minimize a differentiable loss function:

(9) $$\begin{array}{c} \theta_{t+1}\leftarrow \theta_{t}+\alpha \nabla_{\theta }L\left(\theta_{t}\right). \end{array}$$

A robot explores a new environment or exploits the existing information in the learning phase. This is called the *exploration–exploitation* tradeoff [38]. The ϵ-greedy strategy strikes a balance between exploration and exploitation by gradually decreasing the ϵ value from 1.0 to 0.1. The action of a robot is chosen by the ϵ-greedy algorithm using the active Q-network, as shown in the upper part of Figure 2.

Meanwhile, if only a single Q-network is used for learning, there may be oscillation or divergence problems during updating. Therefore, we separated the active (top) and target (bottom) Q-networks, as shown in Figure 2. The learning system is stable by periodically copying the parameters of the active Q-network to the target Q-network during the learning phase [25].

Finally, an *experience replay memory* is introduced to uncorrelate between information [39]; this records experiences $e_{t}=\left({\tilde{s}}_{t},a_{t},r_{t},{\tilde{s}}_{t+1}\right)$ in a replay memory D, and randomly extracts samples for each learning phase.

The pseudo-code for training in an SLE is given in Algorithm 1. First, we initialized the replay memory D, the weights and biases of two neural networks $\theta_{t}^{\prime }$ and $\theta_{t}$ with identical values, and the episode size $n_{ep}$ (line 9–11). Second, we initialized the positions of a robot and an object in $L_{env}\times L_{env}$ space for each episode (line 14). A concatenated new state ${\tilde{s}}_{t}\leftarrow \{s_{t-2},s_{t-1},s_{t}\}$ was also initialized with the identical states (line 15). The ϵ decreases linearly with the ϵ-greedy algorithm (line 16). During the episode, a robot takes a random action $a_{t}$ using the ϵ-greedy algorithm, observes a new state $s_{t+1}$, and then receives a reward $r_{t+1}$ (line 18–19). A new concatenated state ${\tilde{s}}_{t+1}$ at time t+1 is generated from three consecutive frames, and an experience $\left({\tilde{s}}_{t},a_{t},r_{t+1},{\tilde{s}}_{t+1}\right)$ is recorded in the experience replay memory D (line 20–21). This process is repeated $T_{step}$ times until the end of the episode (line 17). In the training phase, we extracted random mini-batch samples from D and it trains $N_{iter}$ times to minimize the loss function $L(s_{t},a_{t}|\theta_{t})$ (line 23–25). The weights of the target Q-network $\theta_{t}^{\prime }$ are substituted with those of the active Q-network $\theta_{t}$ every $T_{K}$ episodes (line 26). Then, we measured the success rate of object transportation to check the current performance (line 27–29). If a robot succeeds in transporting an object to a goal with the success rate $P_{success}$, it stops training at the current level. On the contrary, if the success rate is less than $P_{success}$, it trains more by increasing the unit size of episodes ($N_{ep}$) (line 30–33). When using DRL, there are cases where overtraining can actually hinder learning, and this can be prevented by stopping training after $P_{success}$.
**Algorithm 1:** Training in a standard learning environment
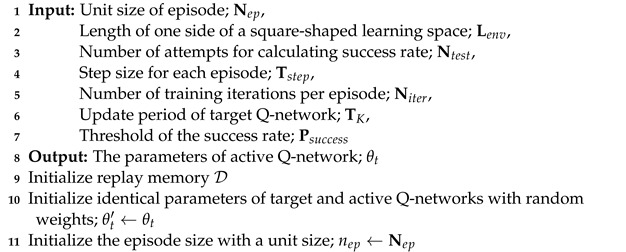

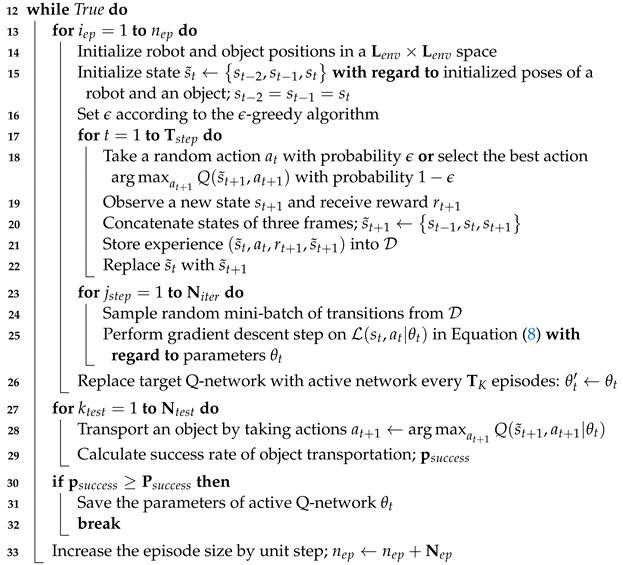


## 6. Object Transportation Using a Task Space Decomposition Method

In the previous section, we presented how a robot could learn how to transport an object in the SLE. While this worked well in the SLE, it was difficult to generalize to larger or more complex environments. In this section, we present a method for transporting objects in complex and large environments using the training results of the SLE. To achieve this, we first generated midpoints, taking into account the inner and outer vertices of the polygonal environment (Section 6.1). Then, we divided a whole task space into several sub-task spaces considering the midpoints and the size of the SLE (Section 6.2). Finally, we computed sub-goals of object transportation for each sub-task space, and the robot can transport the object via the sub-goals (Section 6.3).

### 6.1. Midpoint Generation

**Figure 3:**
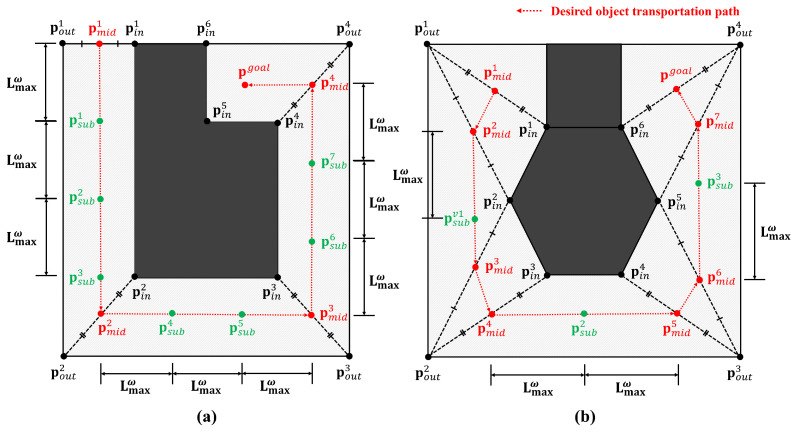
(**a**,**b**) Two examples of midpoints and sub-goal generation using the task space decomposition method.

In order to divide a whole-task space into sub-task spaces, we should first compute the midpoints of the transportation path using sets of the inner and outer vertices in the test environment. Algorithm 2 shows the process of generating midpoints. First, we declared two arrays to hold the distances (dist[][]) and the indices (A[]) between the inner and outer vertices (line 1–2). Second, we computed the Euclidean distances between all inner and outer vertices, and inserted the results into the dist[][] (line 3–5). Third, we computed the outer vertex indices of the minimum distances for each inner vertex and appended them to the A[] (line 6–8). Finally, we computed the midpoints of the inner and outer vertices using the minimum distance pair as $p_{mid}^{k}$ (line 9–11). A list of all midpoints, $P_{mid}$, is returned as the final result (line 12). Figure 3 shows two examples of midpoint generation. A list of midpoints displayed in red dots ($P_{mid}=[p_{mid}^{1},p_{mid}^{2},\ldots ,\;p_{mid}^{N_{mid}}]$) is created by calculating the midpoints of the shortest line segment between the inner and outer vertices.
**Algorithm 2:** Midpoint generation
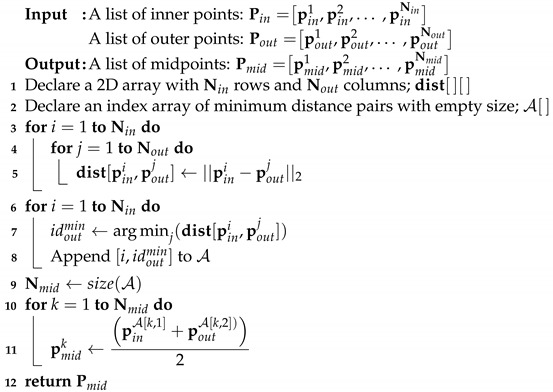


### 6.2. Task Space Decomposition

In general, a whole-task space is larger than the SLE, as already mentioned in Section 5. If the robot is located beyond the size of its SLE, the robot will not know how to transport an object because its state (e.g., the distance from the robot to the object) has not been used for training. Therefore, the whole-task space (i.e., the test environment) should be divided into several sub-task spaces with sizes that are smaller than that of the SLE. This allows a robot to transport an object by following connected lines through midpoints for each sub-task space. However, if a connected line is longer than the size of the SLE, the line should be split into multiple shorter lines. This TSD method is described in detail in Algorithm 3.
**Algorithm 3:** Task space decomposition
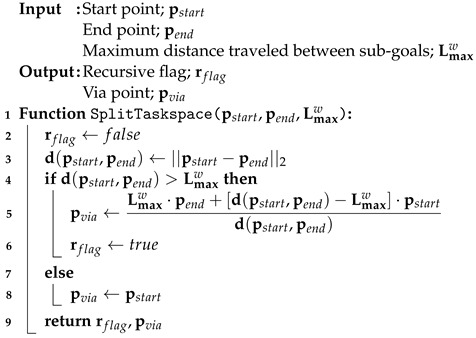


First, the recursive flag $r_{flag}$ is initialized to false and the Euclidean distance between the start and the end points, $d(p_{start},p_{end})$, is calculated (line 2–3). The maximum distance traveled between sub-goals $L_{\max}^{w}$ is equal to $\frac{L_{env}}{2}$ in the SLE because a robot has learned to transport only by the length of $\frac{L_{env}}{2}$. If the distance d is greater than $L_{\max}^{w}$, an intermediate point $p_{via}$ is calculated by dividing $p_{start}$ and $p_{end}$ by $\left[d\left(p_{start},p_{end}\right)-L_{\max}^{w}\right]:L_{\max}^{w}$ (line 5). In this case, the recursive flag is set to true for recursive calculations with respect to the remaining line (line 6). On the other hand, if the distance d is less than $L_{\max}^{w}$, the intermediate point $p_{via}$ ($=p_{start}$) and the recursive flag $r_{flag}$ are returned unchanged (line 8–9).

### 6.3. Sub-Goal Generation and Object Transportation

In the proposed method, a robot can transport an object by sequentially traversing multiple sub-goals. Algorithm 4 shows how to generate a list of sub-goals using the results of Algorithms 2 and 3. Using the SplitTaskspace function in Algorithm 3, we divided a sub-task space for successive midpoints by considering the size of the SLE, $L_{\max}^{w}$ (line 2–3). The starting midpoint $p_{mid}^{id}$ is inserted unconditionally into the list $P_{sub}$ (line 4). If the distance between $p_{via}^{start}$ and $p_{mid}^{id_{new}}$ is greater than $L_{\max}^{w}$, the TSD method is performed recursively until the remaining line is shorter than $L_{\max}^{w}$ (line 5–8). The final output is a list of sub-goals, $P_{sub}$ (line 9).

Now, a robot can transport an object using the Q-network inference result from Algorithm 1 and a list of sub-goals from Algorithm 4. Algorithm 5 shows the object transportation process via sub-goals. First, the index of the sub-goals and the flag are initialized as initial values (line 1–2). A robot observes the state, takes an action to maximize the Q-value from the deep Q-network inference (line 4–6). When an object reaches the sub-goal by robot pushing, the sub-goal index increases by one (line 7–8). When a robot reaches all sub-goals, we consider the object transportation to have succeeded and set the flag to *true* (line 9–10). Finally, the success flag $s_{flag}$ is returned.
**Algorithm 4:** Sub-goal generation for object transportation
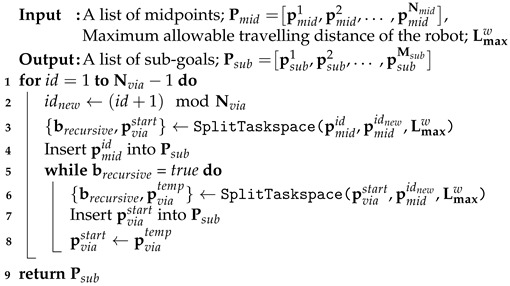


**Algorithm 5:** Object transportation via sub-goals

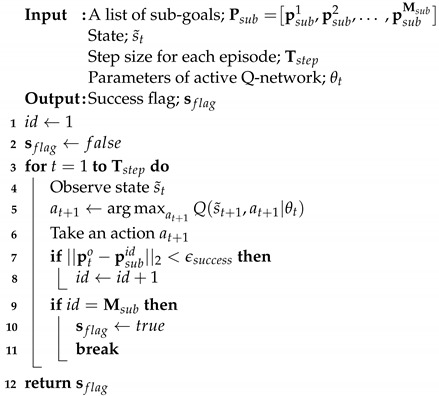



## 7. Simulations

### 7.1. Simulation Environment

We verified the proposed method using a Gazebo simulator based on the ROS [40]. The Gazebo simulator ran 60–80 times faster, and we trained the object transportation model on the Nvidia Geforce GTX-3090 with AMD Ryzen-5950X. In the simulation, the robot was TurtleBot3-waffle [41], and a pallet (1.2 m (W) × 0.8 m (H)) was used as an object. The margins of the goal ($\epsilon_{success}$) and the collision ($\epsilon_{margin}$) in Equation (5) are 0.3 m and 0.1 m, respectively.

The state and action parameters are presented in Table 1. The transitional velocity of 0.3 m/s is the speed at which the robot moves steadily but not too slowly, and the rotational velocity of 1.0 rad/s is the speed at which the robot can adjust its heading in the target direction without turning too sharply when turning from one side to the other, thus compromising the stability of the robot’s motion. The values of the rotational and translational velocities were chosen for the free movement of the robot. The reward of $r_{success}$ is 20 times larger than $r_{collision}$ in absolute value. We also gave more weight to the distance difference between the goal–object than the robot–object. This is because completing the object transportation is the most important mission; it does not matter if the robot arrives at the goal or not.

The hyperparameters of the training phase are shown in Table 2. These hyperparameters were chosen to quickly and efficiently learn the object transportation method in the SLE. In particular, we varied the length of one side of the SLE to 6, 8, and 12 m. In order to compare the performance of different training space sizes, we ran the training in different SLE. In addition, the threshold of the success rate $P_{success}$ was set to 0.98 to guarantee the minimum performance of object transportation. The learning capacity of the Q-network is related to the depth and width of the layers, and thus we chose a depth and width of 4 and 256, respectively.

### 7.2. Training Results in Standard Learning Environments

We trained the object transportation method based on DRL in SLEs of different sizes: 6×6 m, 8×8 m, and 12×12 m. The goal position was fixed to the origin, and the position of the robot was randomly initialized for each episode. If the current success rate $p_{success}$ is lower than the success threshold ($P_{success}=0.98$), we increased the episode by one unit step ($N_{ep}=500$) until the current success rate reached the threshold, as already mentioned in Algorithm 1. The final training results are described in Table 3. The success rates were higher than the success rate threshold for all environments. In addition, the number of episodes needed to reach the success rate threshold increased as the size of the environment increased. This is because a robot should learn more in the large environment.

**Figure 4:**
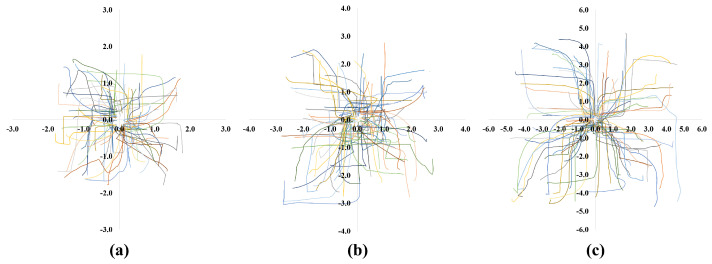
One hundred transportation trajectories of an object in different sizes of SLEs: (**a**) 6×6 m environment; (**b**) 8×8 m environment; (**c**) 12×12 m environment.

Figure 4 shows 100 trajectories of an object in different SLEs. The robot successfully learned how to transport an object in SLEs regardless of the initial positions of the robot and an object. This result will be used for transporting an object in different test environments, which will be discussed in the next section.

### 7.3. Test Results in Various-Shaped Environments

**Figure 5:**
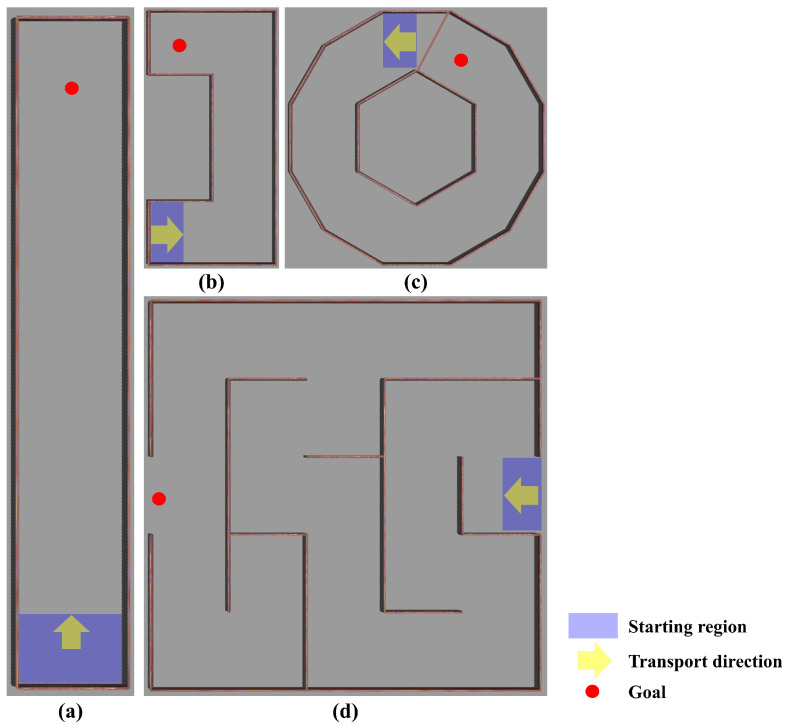
Various simulation environments for the testing of the proposed method: (**a**) long corridor; (**b**) simple polygon; (**c**) complex polygon; (**d**) maze.

In the training phase, the SLEs are square environments of different sizes. These environments are designed to facilitate optimization and to improve convergence in the training phase. However, in the real world, there are many different sizes and shapes of environments. Therefore, we built our test environment with the following two aspects in mind: (1) sub-goal changes and (2) path length. For example, if a robot transports along a straight line, the path length is long, but the sub-goal changes are infrequent. Conversely, in a maze, the length of the segmented path is typically short, but the sub-goals change frequently and sequentially as the object is transported. In the real world, sub-goal changes and path length changes occur frequently, and thus the following environments are constructed to reflect this, as shown in Figure 5: (a) a long corridor; (b) a simple polygon; (c) a complex polygon; (d) a maze-shaped environment.

Table 4 shows the end-to-end DRL (i.e., the existing method) results in the differently shaped environments. The robot can be trained without any pre-processing, such as task decomposition, which is called end-to-end DRL. While the end-to-end DRL-based object transportation methods are easy to adapt to a wide variety of environments, they perform poorly in complex environments, as shown in Table 4; there are some successes with relatively simple environments, such as long corridors, but the robot cannot transport objects in complex shapes, such as polygons and mazes. However, in the case of end-to-end learning, we found that the transport distance is relatively short because we did not specify any waypoints.

**Figure 6:**
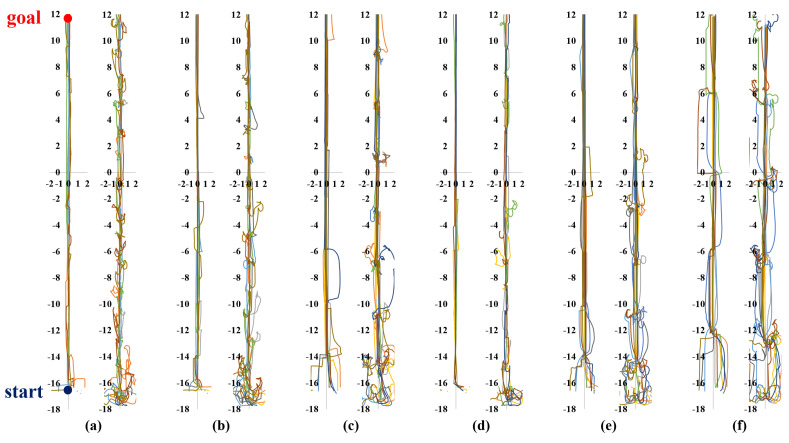
Ten trajectories of object transportation in a long corridor environment (Figure 5a). The left and right sides of each figure represent the trajectory of an object and a robot, respectively. (**a**) $L_{env}=6$ m and $L_{\max}^{w}=2$ m; (**b**) $L_{env}=8$ m and $L_{\max}^{w}=2$ m; (**c**) $L_{env}=8$ m and $L_{\max}^{w}=4$ m; (**d**) $L_{env}=12$ m and $L_{\max}^{w}=2$ m; (**e**) $L_{env}=12$ m and $L_{\max}^{w}=4$ m; (**f**) $L_{env}=12$ m and $L_{\max}^{w}=6$ m.

**Figure 7:**
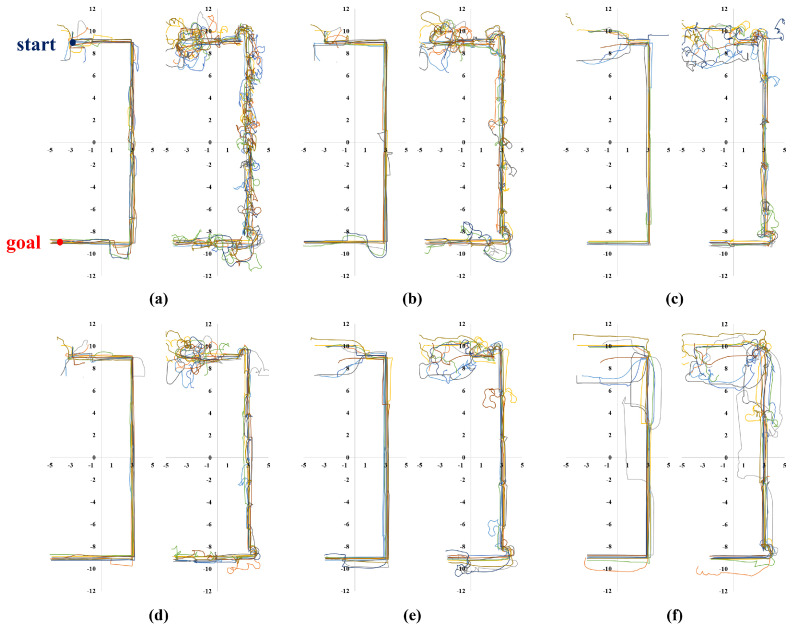
Ten trajectories of object transportation in a simple polygon environment (Figure 5b). The left and right sides of each figure represent the trajectory of an object and a robot, respectively. (**a**) $L_{env}=6$ m and $L_{\max}^{w}=2$ m; (**b**) $L_{env}=8$ m and $L_{\max}^{w}=2$ m; (**c**) $L_{env}=8$ m and $L_{\max}^{w}=4$ m; (**d**) $L_{env}=12$ m and $L_{\max}^{w}=2$ m; (**e**) $L_{env}=12$ m and $L_{\max}^{w}=4$ m; (**f**) $L_{env}=12$ m and $L_{\max}^{w}=6$ m.

**Figure 8:**
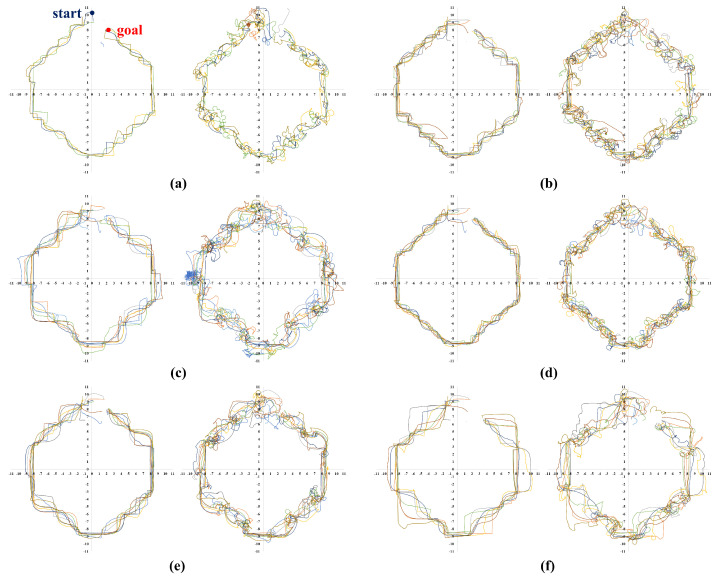
Ten trajectories of object transportation in a complex polygon environment (Figure 5c). The left and right sides of each figure represent the trajectory of an object and a robot, respectively. (**a**) $L_{env}=6$ m and $L_{\max}^{w}=2$ m; (**b**) $L_{env}=8$ m and $L_{\max}^{w}=2$ m; (**c**) $L_{env}=8$ m and $L_{\max}^{w}=4$ m; (**d**) $L_{env}=12$ m and $L_{\max}^{w}=2$ m; (**e**) $L_{env}=12$ m and $L_{\max}^{w}=4$ m; (**f**) $L_{env}=12$ m and $L_{\max}^{w}=6$ m.

**Figure 9:**
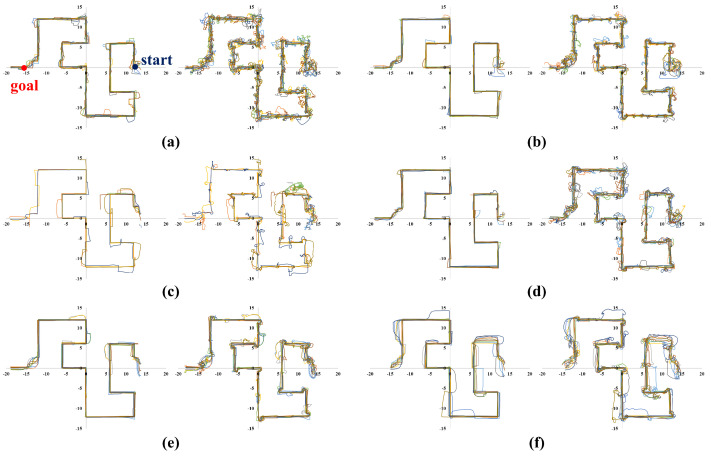
Ten trajectories of object transportation in a maze environment (Figure 5d). The left and right sides of each figure represent the trajectory of an object and a robot, respectively. (**a**) $L_{env}=6$ m and $L_{\max}^{w}=2$ m; (**b**) $L_{env}=8$ m and $L_{\max}^{w}=2$ m; (**c**) $L_{env}=8$ m and $L_{\max}^{w}=4$ m; (**d**) $L_{env}=12$ m and $L_{\max}^{w}=2$ m; (**e**) $L_{env}=12$ m and $L_{\max}^{w}=4$ m; (**f**) $L_{env}=12$ m and $L_{\max}^{w}=6$ m.

Meanwhile, Figure 6, Figure 7, Figure 8 and Figure 9 show representative trajectories of an object and a robot using the proposed method for each test environment; only 10 trajectories out of a total of 200 trials are shown for easy comparison between trajectories. The left and right sides of each figure show the trajectories of an object and a robot, respectively. By comparing (a), (b), (d) or (c), (e) for each figure, we can see that the larger the size of the SLE, the less volatile the desired trajectory of the robot and the object. This means that if a robot learns how to transport in a large learning environment, the motion variations in the test environment are small. Meanwhile, for the same SLE size, the smaller the maximum distance traveled between sub-goals, the more likely the robot is to travel directly to the goal without motion variation. This is because the larger the maximum distance between sub-goals, the longer the object will stay in its current pushing direction once it starts pushing, making it harder to change the pushing direction; we can derive these conclusions by comparing (b), (c) or (d), (e), (f) for each figure. In general, it is a good result if the trajectory is plotted consistently and without deviation over several trials. For example, we can see that the case of Figure 6d has less path variability than that of (f). This indicates that Figure 6d performs better than (f) in that it is able to transport the object reliably every time.

Table 5 shows the test results in differently shaped environments using the proposed method. We tested 200 trials in four test environments using the deep Q-network learned from the differently sized SLEs. The average distance traveled by the robot and the object was recorded only if the object transportation was succeeded.

From the table, we can see the following results. First, we can see that the success rate is generally higher for 12×12 m. This is because the robot can also transport an object in a small environment if it has learned on a large environment. However, as shown in Table 3, the larger the training environment, the more episodes needed for training, which could lead to long training times or show poor convergence. Therefore, we should design the size of the SLE appropriately by considering the trade-off between the performance and training time. Second, the success rate increases as the maximum distance traveled between sub-goals increases. Conversely, the smaller the distance between sub-goals, the greater the risk that the object transportation will fail. This is because the greater the number of sub-goals, the more motion changes that are required to reach the sub-goals; frequent motion changes make it difficult for a robot to control precisely.

## 8. Discussion

In general, DRL-based object transportation has the advantage of it being easy to learn pushing motions, but it has the disadvantage of it being difficult to apply in various environments; this is because the DRL algorithm only works well within the specific conditions and environment in which it has been trained. Therefore, we solved this problem by using the task space decomposition method while maintaining the advantages of the DRL algorithm’s ease of learning how to transport an object.

Furthermore, the proposed method has shown that a robot can transport an object not only in simple environments but also in complex environments. A robot learns how to transport an object on short paths in simple environments (e.g., a square environment) and extends these results to set up multiple sub-goals to apply to larger, more complex environments, such as a long corridor, polygon, and maze. Finally, the robot transports the object by passing through the set sub-goals in sequence. In this way, it is possible to transport objects in unknown and arbitrary environments, not just the environment in which they were trained; the proposed method will be useful in the real world, where complex structures and environments exist.

On the other hand, the proposed method has some limitations. First, objects have to be transported via sub-goals, which may lead to inefficient routes. Inefficient energy consumption may occur because the minimum distance is not considered in the process of setting sub-goals. Therefore, it is necessary to consider the efficiency of the route at the sub-goal selection stage for optimal object transport. Second, depending on the environmental conditions, it may not be possible to transport with a single robot. Due to the shape of the object and the surrounding environment, there is a possibility of falling into a local minimum, so a transport solution may not exist with a single robot. In this case, a multi-robot object transport method should be considered.

## 9. Conclusions

This paper proposed a novel DRL-based object transportation using a task space decomposition method. First, a robot learned how to transport an object in the SLE based on DRL. Second, a whole-task space was divided into several sub-task spaces using the task space decomposition method. The learning results from the SLE can be applied to each sub-task space without modification. Finally, the robot can transport an object in arbitrary environments as well as in the SLE; the performance of the existing DRL-based object transportation method dropped dramatically when the test environment was different from the training environment or in large and complex environments. On the other hand, since our proposed method decomposes the whole environment into simple and easy environment units to transport objects, we found that it works well in arbitrary environments such as a long corridor, polygon, and maze. In the future work, we will consider various environments with static and dynamic obstacles, such as a logistics and airport environment. We will also verify the performance of the proposed algorithm on a real robot.

## Figures and Tables

**Table 1:** State and action parameters of the proposed object transportation.

Category	Description	Index	Value
Action (Equation (4))	Rotational velocity	$v_{rot}$	1.0 rad/s
	Translational velocity	$v_{trans}$	0.3 m/s
Reward (Equation (5))	Success reward	$r_{success}$	1.0
	Collision reward	$r_{collision}$	−0.05
	Weight	$w_{1}$	1.0
		$w_{2}$	0.1

**Table 2:** Hyperparameters of the training phase.

Source	Description	Index	Value
Training (Algorithm 1)	Unit size of episode	$N_{ep}$	500
	Length of one side of the SLE	$L_{env}$	6/8/12 m
	Number of attempts for calculating success rate	$N_{test}$	30
	Step size for each episode	$T_{step}$	1000
	Number of training iterations per episode	$N_{iter}$	200
	Update period of target Q-network	$T_{K}$	32
	Threshold of the success rate	$P_{success}$	0.98
Q-Network (Figure 2)	Depth of Q-network	-	4
	Width of each layer	-	256
	Activation function	-	ReLU
	Learning rate	α	0.001
	Discount factor	γ	0.99
	Batch size	-	512
	Replay memory size	# of D	$1.0\times 10^{7}$
	Initial exploration probability in ϵ-greedy	-	1.0
	Final exploration probability in ϵ-greedy	-	0.1

**Table 3:** Results in the standard learning environment.

Environment Size ($L_{\mathrm{env}}\times L_{\mathrm{env}}$)	The Number of Success/Trial	Success Rate ($p_{\mathrm{success}}$)	The Number of Training Episodes ($n_{\mathrm{ep}}$)
6×6 m	100/100	100%	2000
8×8 m	99/100	99%	4000
12×12 m	100/100	100%	8000

**Table 4:** Results in the end-to-end deep reinforcement learning method (the existing method).

Environment	The Number of Success/Trial (Success Rate)	Avg. Travelling Distance of an Object	Avg. Travelling Distance of a Robot
Long corridor (Figure 5a)	133/200 (0.67)	23.59 m	23.21 m
Simple polygon (Figure 5b)	0/200 (0.0)	-	-
Complex polygon (Figure 5c)	0/200 (0.0)	-	-
Maze (Figure 5d)	0/200 (0.0)	-	-

**Table 5:** Test results in differently shaped environments.

Environment	Size of Standard Learning Environment ($L_{\mathrm{env}}\times L_{\mathrm{env}}$)	Maximum Distance Traveled between Sub-Goals ($L_{\max}^{w}$)	The Number of Success/Trial (Success Rate)	Avg. Travelling Distance of an Object	Avg. Travelling Distance of a Robot
Long corridor (Figure 5a)	6 m × 6 m	2 m	127/200 (0.64)	37.47 m	36.42 m
	8 m × 8 m	2 m	143/200 (0.71)	29.55 m	28.86 m
		4 m	193/200 (0.96)	26.94 m	26.02 m
	12 m × 12 m	2 m	131/200 (0.66)	27.82 m	27.82 m
		4 m	183/200 (0.92)	20.20 m	19.75 m
		6 m	**200/200 (1.00)**	19.93 m	19.33 m
Simple polygon (Figure 5b)	6 m × 6 m	2 m	**196/200 (0.98)**	27.45 m	27.00 m
	8 m × 8 m	2 m	**196/200 (0.98)**	22.61 m	22.25 m
		4 m	146/200 (0.73)	22.25 m	22.39 m
	12 m × 12 m	2 m	190/200 (0.95)	21.32 m	21.11 m
		4 m	195/200 (0.97)	17.15 m	16.75 m
		6 m	191/200 (0.95)	18.08 m	17.88 m
Complex polygon (Figure 5c)	6 m×6 m	2 m	150/200 (0.75)	59.97 m	61.67 m
	8 m × 8 m	2 m	166/200 (0.83)	59.53 m	61.10 m
		4 m	166/200 (0.83)	56.72 m	57.46 m
	12 m × 12 m	2 m	**179/200 (0.90)**	54.33 m	55.47 m
		4 m	177/200 (0.89)	47.75 m	48.78 m
		6 m	157/200 (0.79)	56.71 m	57.14 m
Maze (Figure 5d)	6 m × 6 m	2 m	192/200 (0.96)	151.06 m	150.05 m
	8 m × 8 m	2 m	198/200 (0.99)	125.18 m	124.08 m
		4 m	135/200 (0.68)	134.40 m	133.41 m
	12 m × 12 m	2 m	**200/200 (1.00)**	116.70 m	115.58 m
		4 m	**200/200 (1.00)**	102.38 m	101.46 m
		6 m	196/200 (0.98)	98.57 m	97.91 m

## Data Availability

Not applicable.

## Abbreviations

The following abbreviations are used in this manuscript:

RL	Reinforcement learning
DRL	Deep reinforcement learning
SLE	Standard learning environment
TSD	Task space decomposition
DQN	Deep Q-network
